# Supplementary material for: Divergent evolution of slip banding in CrCoNi alloys
Source: Nat Commun. 2025 Apr 16;16:3631. doi: 10.1038/s41467-025-58480-4 (PMC12003725; doi:10.1038/s41467-025-58480-4)
Supplement: Supplementary file 1 — Supplementary Information [file 41467_2025_58480_MOESM1_ESM.pdf]

Supplementary Information

Divergent Evolution of Slip Banding in CrCoNi Alloys

Bijun Xie[1,†], Hangman Chen[1,†], Pengfei Wang[1], Cheng Zhang[2], Bin Xing[2], Mingjie Xu[2], Xin Wang[2], Lorenzo Valdevit[1,2], Julian Rimoli[1], Xiaoqing Pan[2], Penghui Cao[1,2,*]

[1]Department of Mechanical and Aerospace Engineering, University of California, Irvine, CA 92697, United States.

[2]Department of Material Science and Engineering, University of California, Irvine, California 92697, United States

Table of Contents

Supplementary Figures 1-21 p. 2

Supplementary Tables 1-2 p. 22

Supplementary Note 1-4 p. 25

Supplementary References p. 28

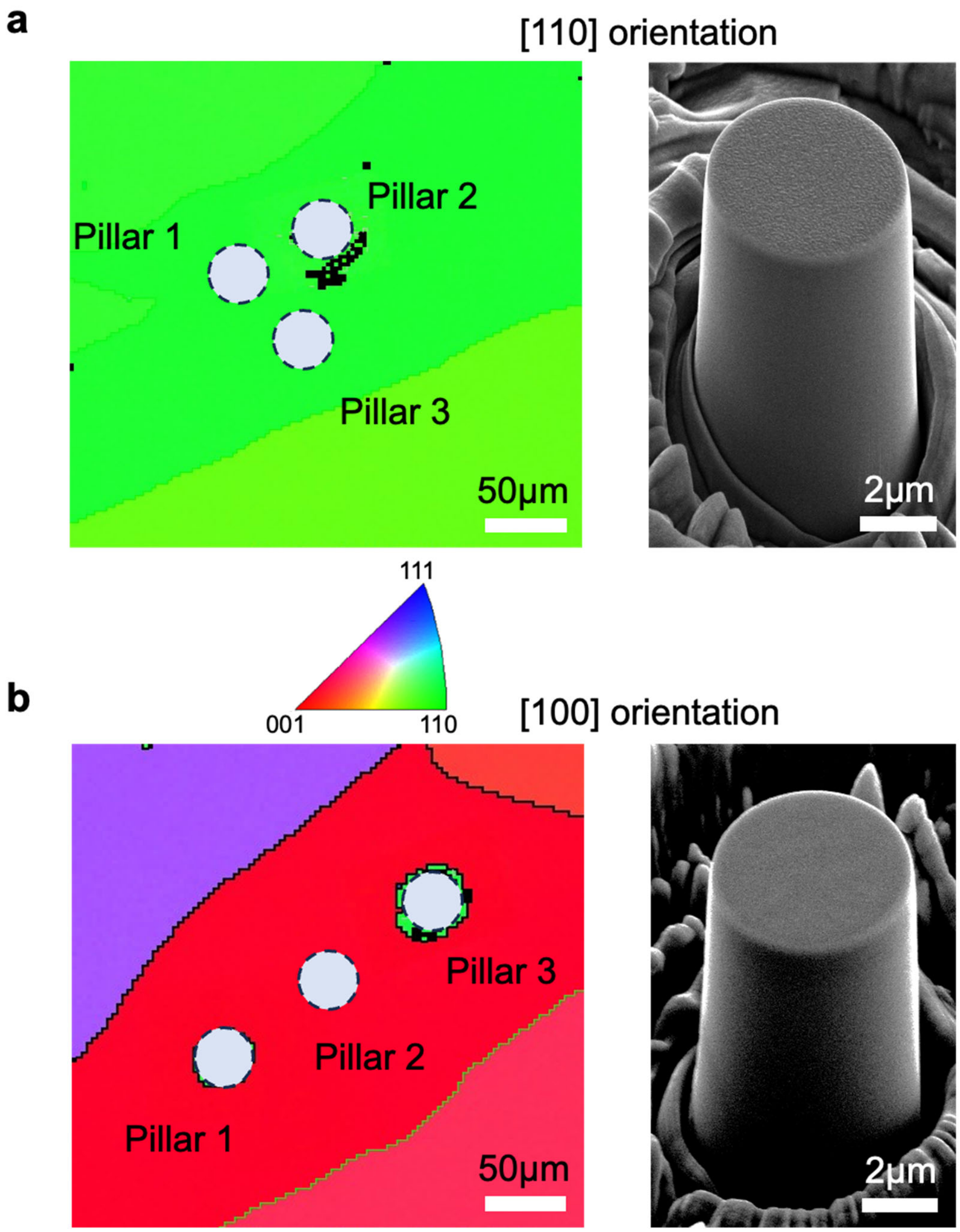

Supplementary Fig. 1 | EBSD maps showing crystallographic orientations of polycrystals and the single-crystal micropillar fabricated within targeted grains in the quenched CrCoNi MEA. a, represents the fabrication of [110]-oriented micropillars. **b**, shows the [100]-oriented micropillars.

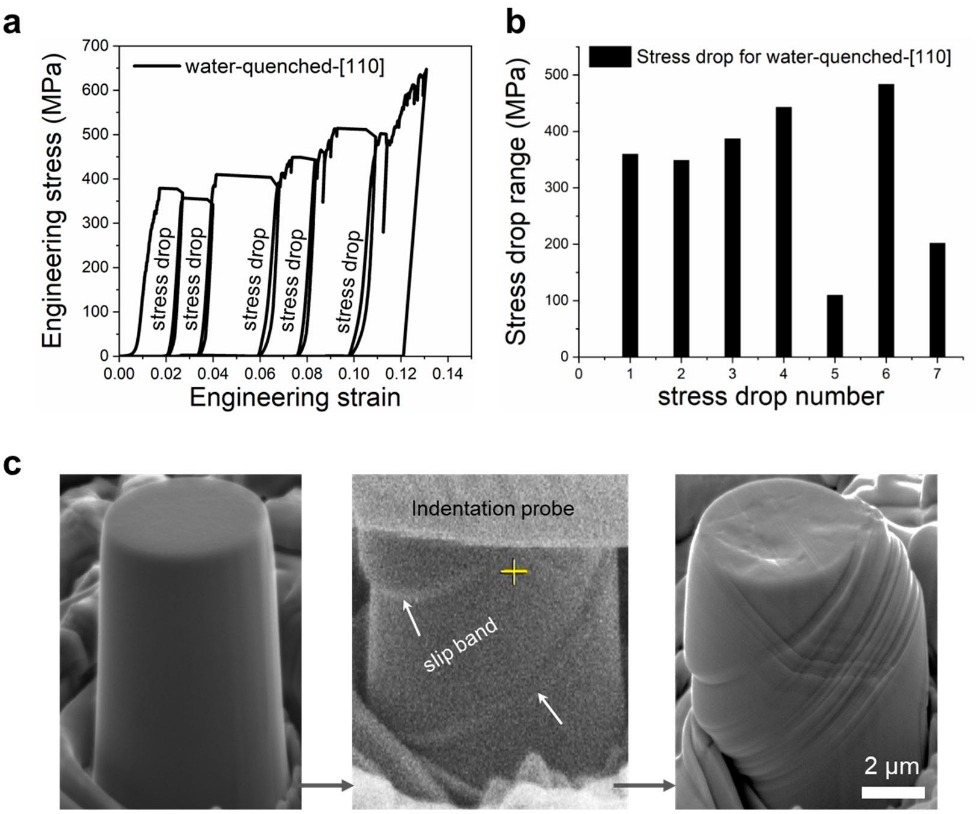

Supplementary Fig. 2 | In-situ SEM compression test on [110]-oriented micropillar. a, Engineering stress-strain curve showing serration and large stress drop events (avalanches) in plastic flow regime. **b**, Statistic analysis of large events. The stress drop sizes ranges from ~100 to ~500 MPa. **c**, The formation of sharp surface steps and confined slip bands under compression. Surface steps are marked by the white arrows.

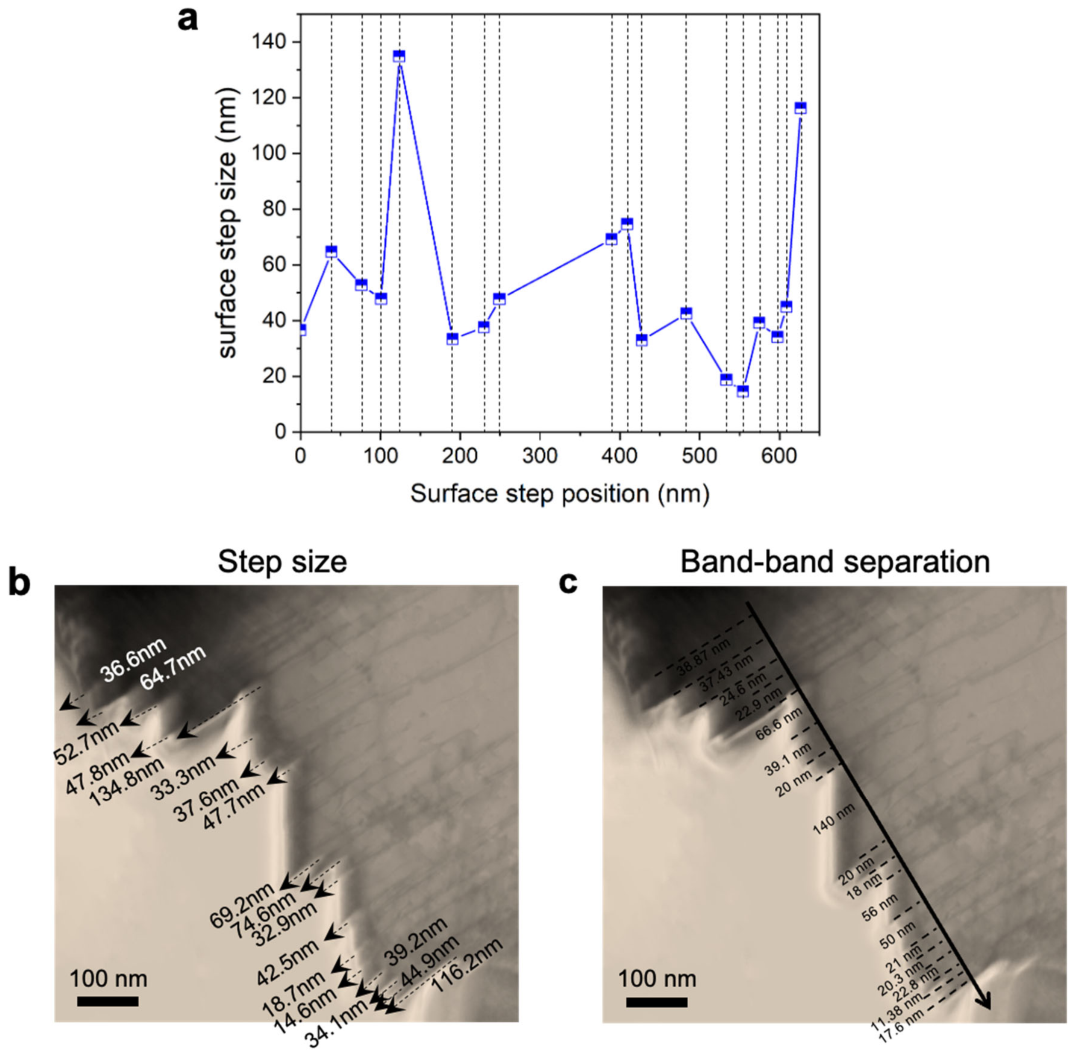

Supplementary Fig. 3 | Deformation surface morphology in [110]-oriented micropillar. a, The surface step size and its location. **b**, Sharp surface steps, corresponding to C-SBs, have a size approximately ranging from 14 nm to 134.8 nm. **c**, Band-band separation is irregular, and approximately ranges from 11.38 nm to 140 nm.

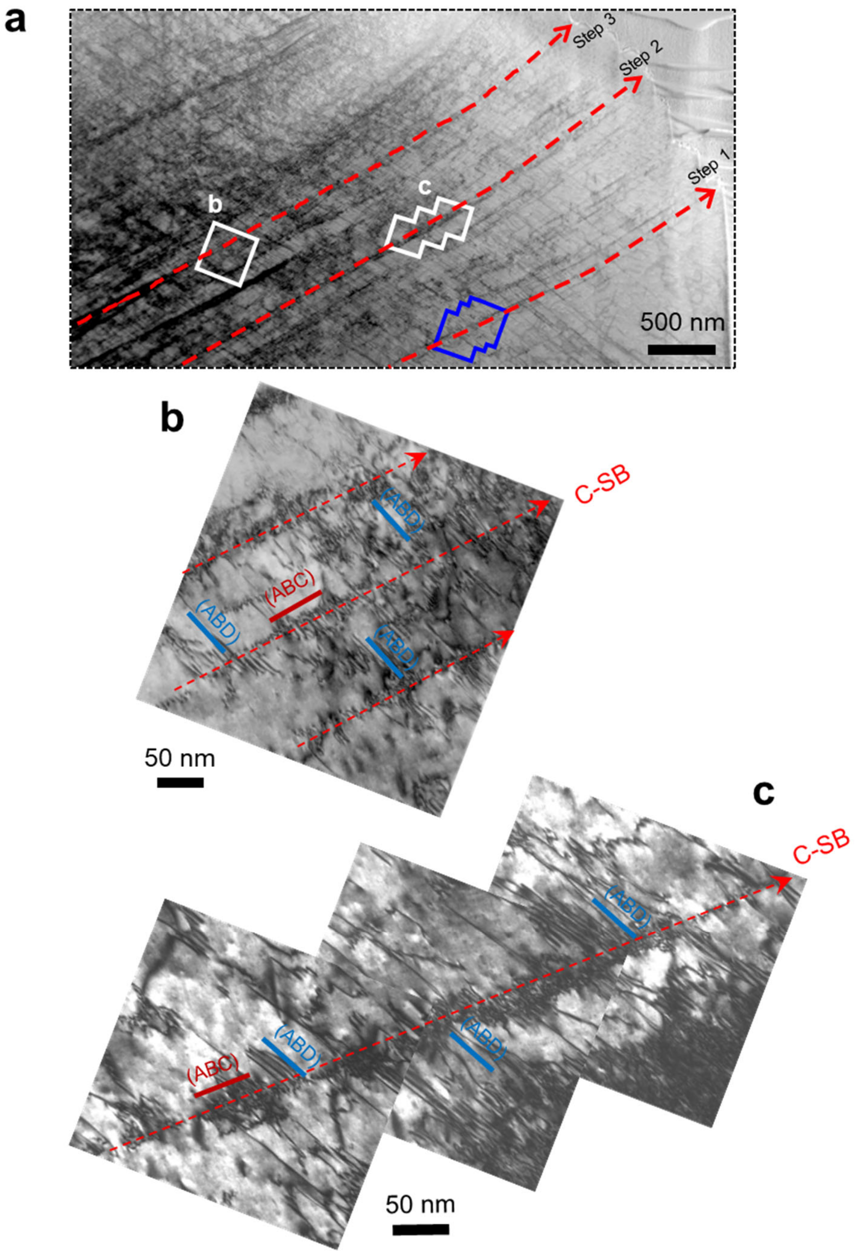

Supplementary Fig. 4 | Microstructural features of C-SBs. a, BF-STEM image of the plastically deformed regime in the pillar. **b** and **c**, Enlarged BF-TEM images of the C-SBs as indicated in (**a**), showing the structures of C-SBs with a dense array of SFs along the secondary slip plane, ABD.

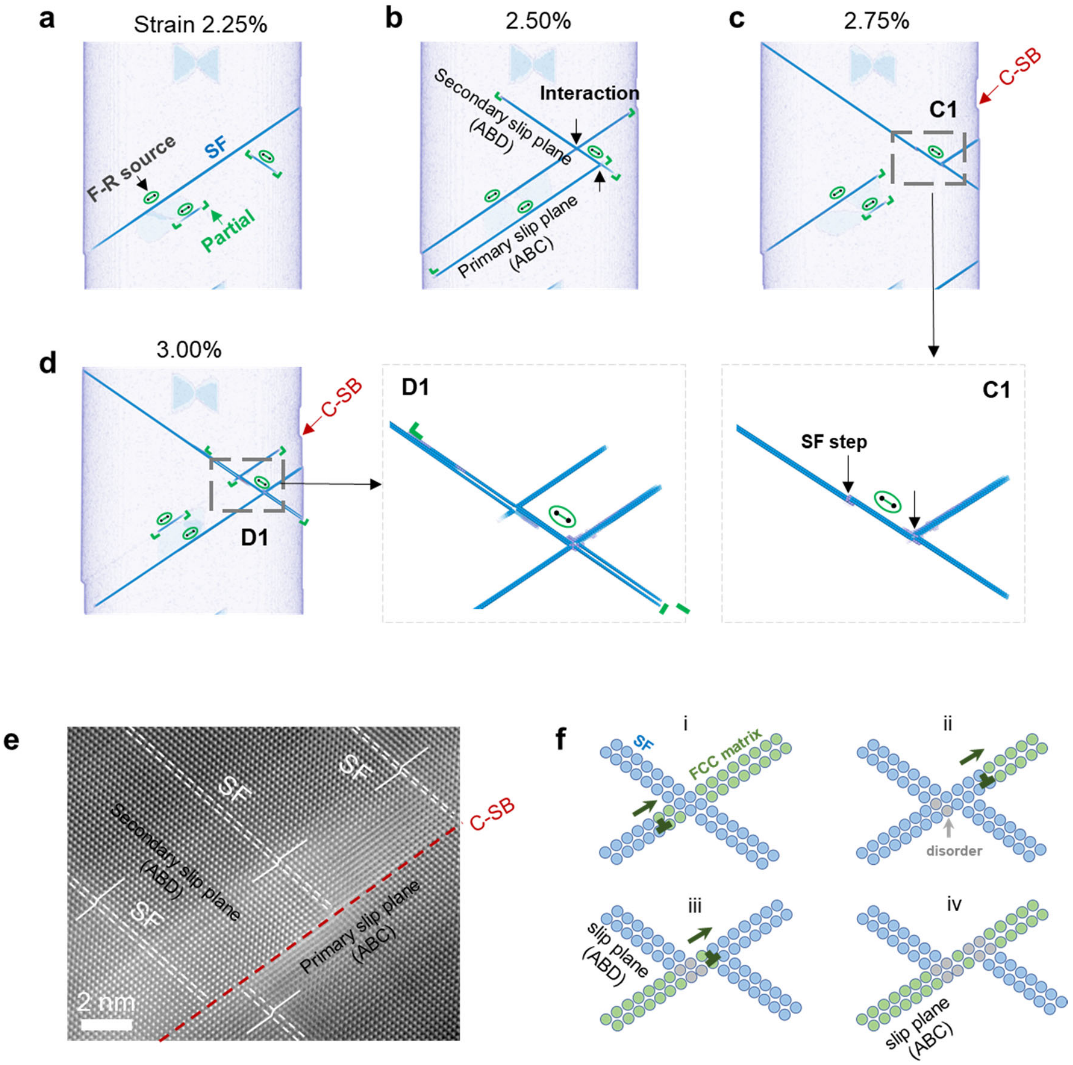

Supplementary Fig. 5 | Formation mechanisms of the stepped SFs on secondary slip plane. **a-d**, Dislocations emit from Frank-Read sources marked by green circles. The formed SFs on the secondary slip plane (ABD) are displaced by a magnitude of one Burgers vector after passage of a dislocation on the primary slip plane. **e**, HAADF-STEM image of C-SB region shows the stepped SFs on the ABD plane intersecting with the primary slip plane ABC. **f**, Schematic illustration of the stepped SF formation due to passing through a pair of partial dislocations.

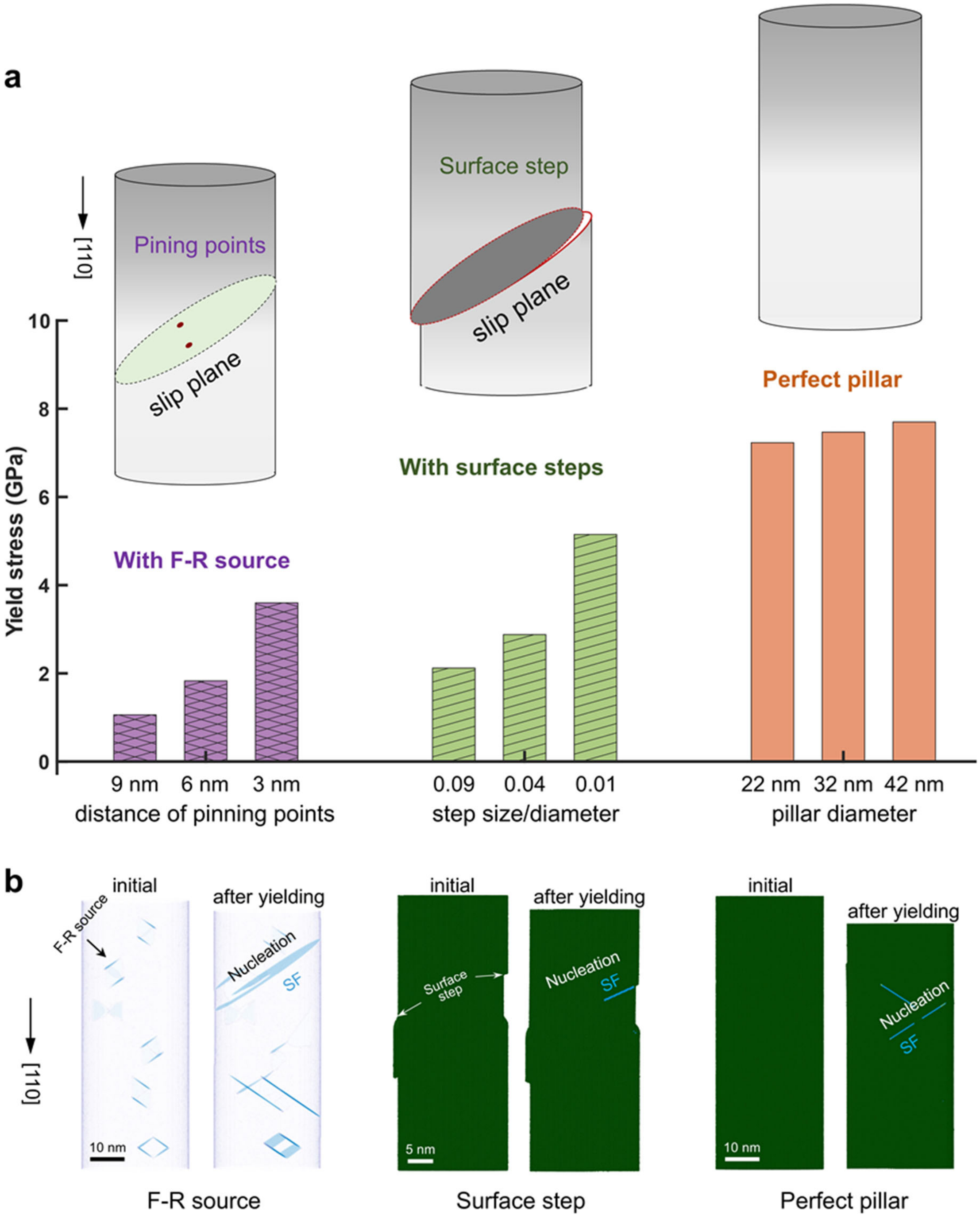

Supplementary Fig. 6 | Dislocation nucleation stress comparison at Frank-Read (F-R) source, surface step, and free surface. a, Variation of yield stress with pinning point distance (F-R source), surface step size, and pillar diameter, for loading in [110] direction. **b**, Corresponding structures post-dislocation emission (i.e., after yielding). For pillars with Frank-Read source, only non-fcc atoms are displayed to show the slip initiated from sample interior.

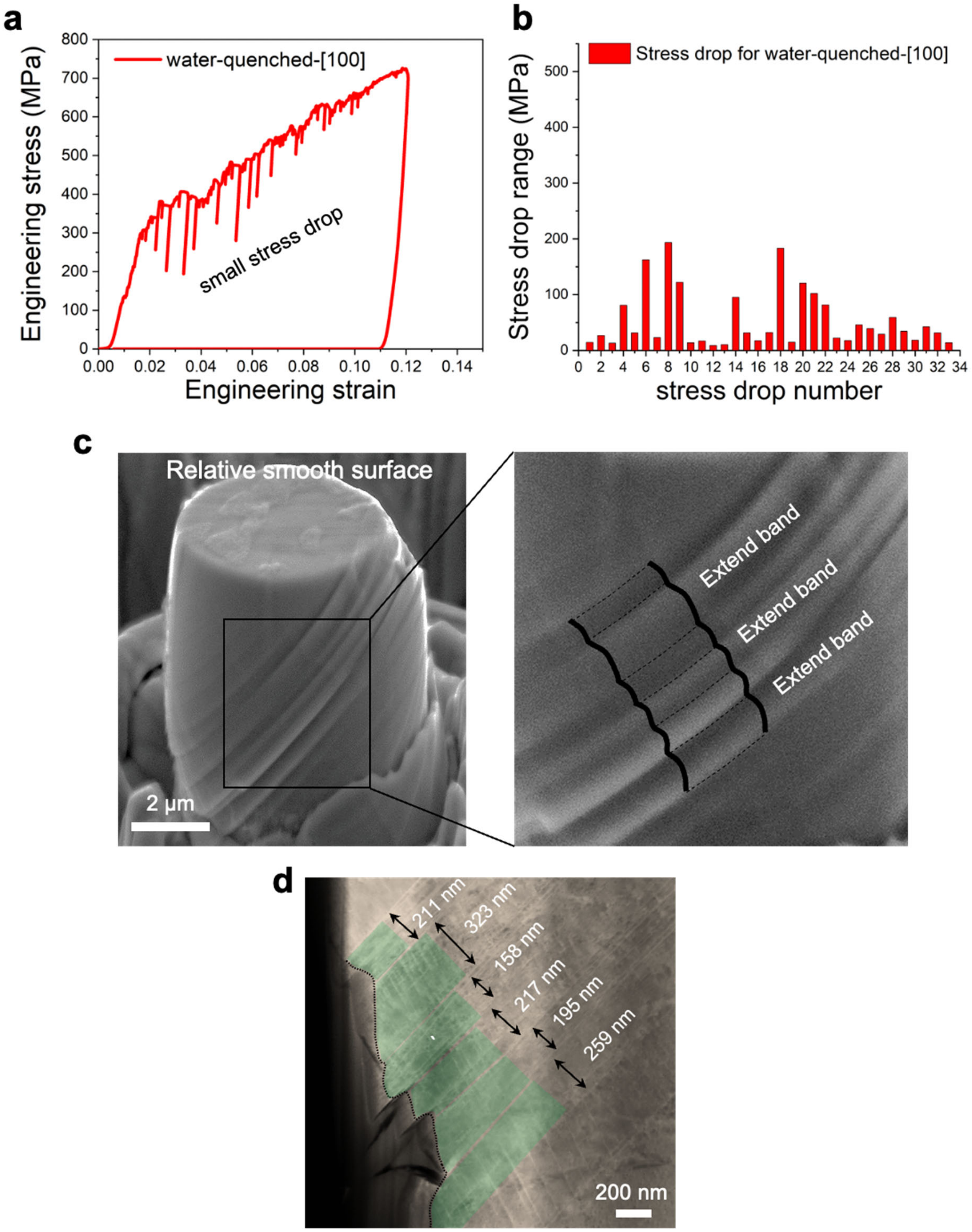

Supplementary Fig. 7 | In-situ compression test on [100]-oriented micropillar. a, Engineering stress-strain curve for uniaxial compression showing that the large stress drop events are diminished during plastic flow. **b**, Statistic analysis of stress drop of stress-strain curve in (**a**), showing relatively small stress drops. **c,** SEM images of surface morphology showing smooth surface and extended bands. **d**, HAADF-STEM image of deformation surface reveals extended bands, with thickness from 158 nm to 323 nm.

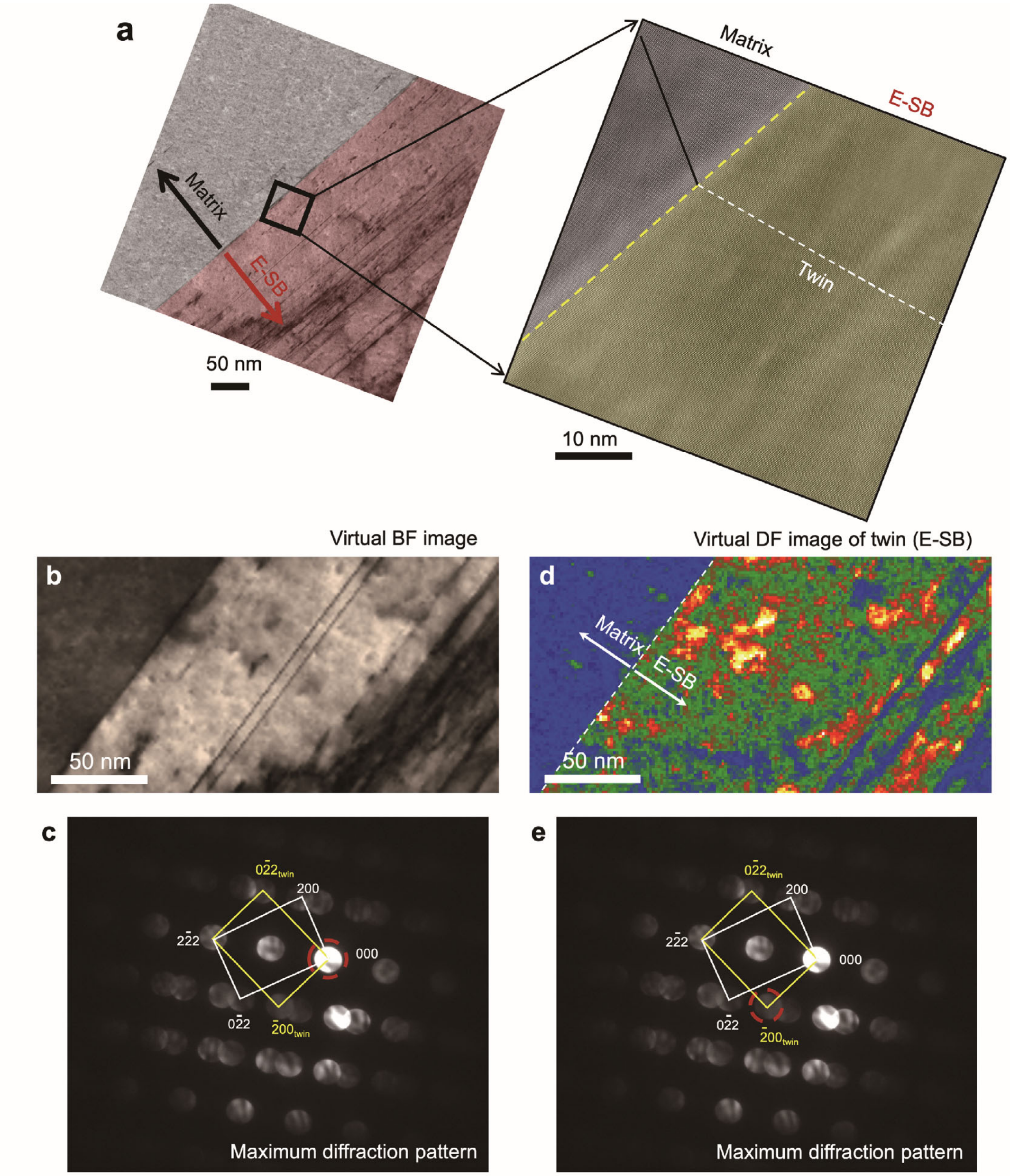

Supplementary Fig. 8 | Microstructures of E-SB. a, The interfacial regions between the un-plastically deformed matrix and the E-SB, showing large-size deformation twin. The left panel presents BF-STEM image and the right panel provides atomic-resolution HAADF-STEM image. **b-e**, 4D-STEM characterization of the E-SBs confirming the extended deformation twin. **b,** Virtual BF image constructed from the 4D-STEM diffraction. The virtual aperture was placed at the (000) beam, as indicated by the red circle in (**c**). **d**, Virtual dark-field image using a [$\bar{2}$00] twin reflection as shown with the red circle in (**e**).

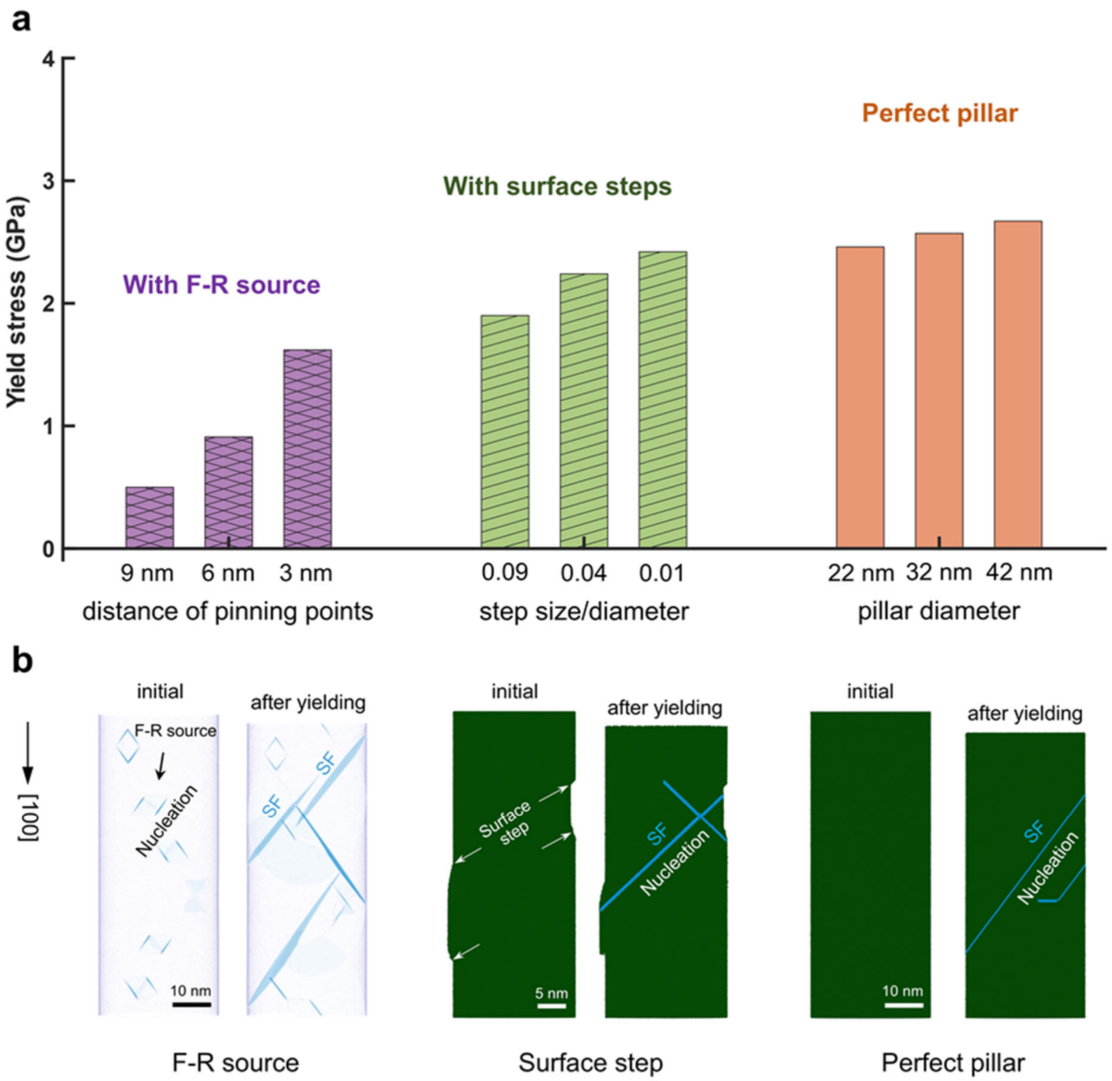

Supplementary Fig. 9 | Dislocation nucleation stress comparison at Frank-Read source, surface step, and free surface. a, Variation of yield stress with pinning point distance (F-R source), surface step size, and pillar diameter, for loading in [100] direction. **b**, Corresponding structures post-dislocation emission (i.e., after yielding). For pillars with Frank-Read source, only non-fcc atoms are displayed to show the slip initiated from sample interior.

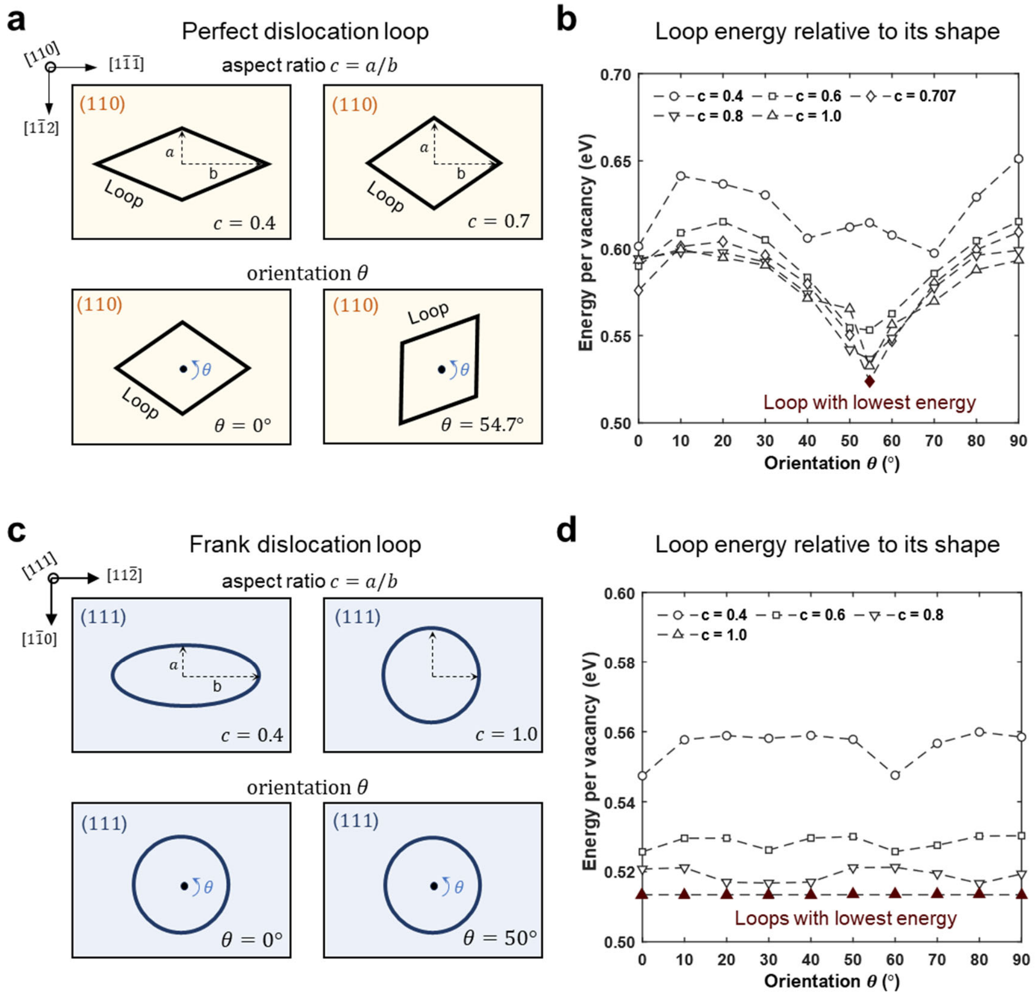

Supplementary Fig. 10 | Stable vacancy-type dislocation loops. The two primary types of loops in fcc considered are the rhombic 1/2<110>{110} perfect loop and the 1/3<111>{111} Frank loop. **a**, Schematic illustration of rhombic perfect loop shape with varying aspect ratio and orientation, and **b**, formation energy relative to its shape. **c**, Illustration of Frank loop with different aspect ration and orientation, and **d**, its formation energies for different shapes.

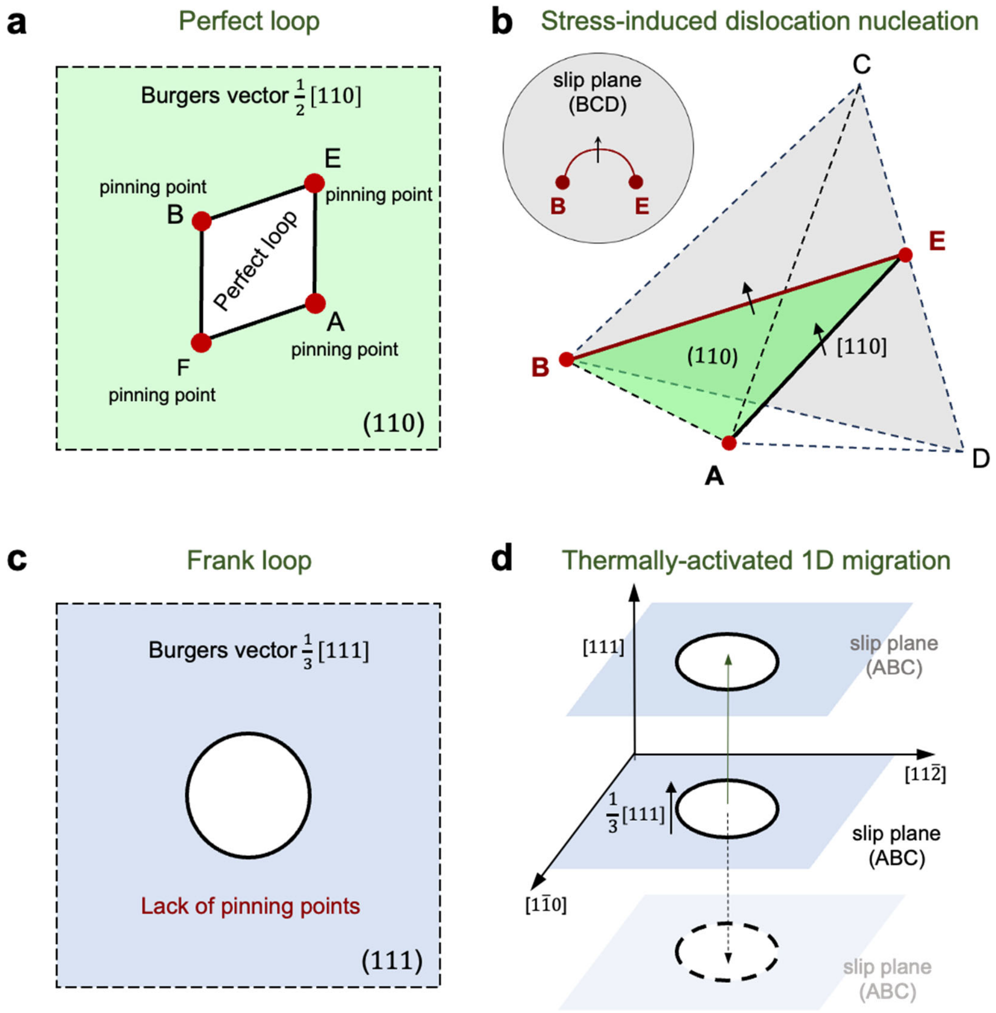

Supplementary Fig. 11 | Dislocation nucleation and migration mechanisms in the two types of loops. **a**, A perfect rhombic loop located on the (110) plane, with the vertices of the loop acting as pinning points. **b**, The relative position of the perfect loop with Thompson tetrahedron. Under shear stress, dislocation nucleation can occur. For instance, the BE segment of the loop lies on the BCD slip plane and carries the full Burgers vector of 1/2[110]. This anchored dislocation line serves as an effective nucleation site (Frank-Read source) under shear stress. **c**, A Frank loop locates on (111) plane with Burgers vector 1/3[111]. Lacking pinning points, it cannot emit dislocations. Instead, this loop typically undergoes one-dimensional migration driven by thermal activation, making it insensitive to mechanical stress.

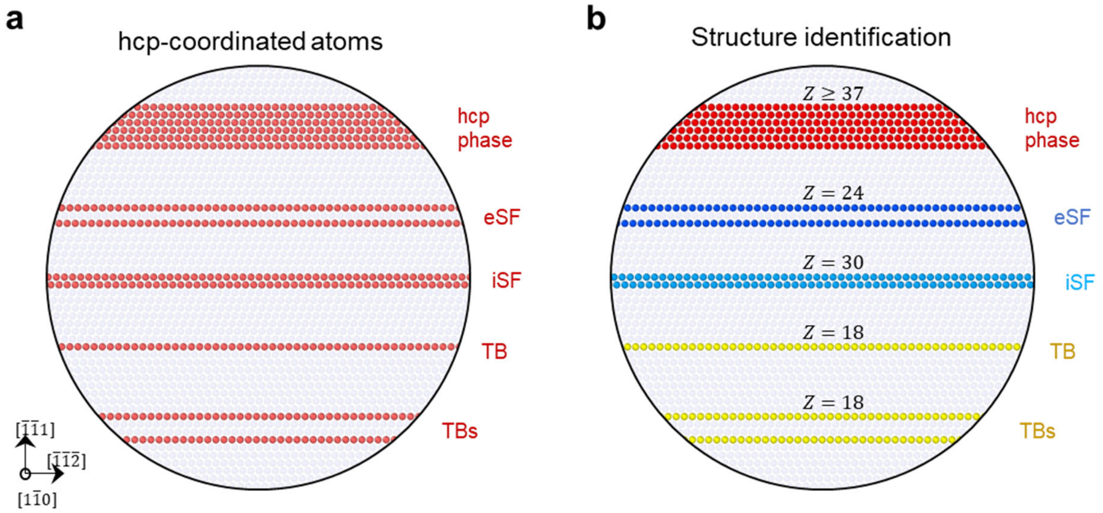

Supplementary Fig. 12 | Deformation hcp phase, SF, and twin boundary identification in atomistic simulations. **a**, A fcc structure containing hcp-coordinated atoms. **b**, All the hcp structures, including hcp phase, intrinsic SF (iSF), extrinsic SF (eSF) and TB are identified from Z ($Z_{hcp} \geq 37, Z_{eSF} = 24, Z_{iSF} = 30, Z_{TB} = 18$).

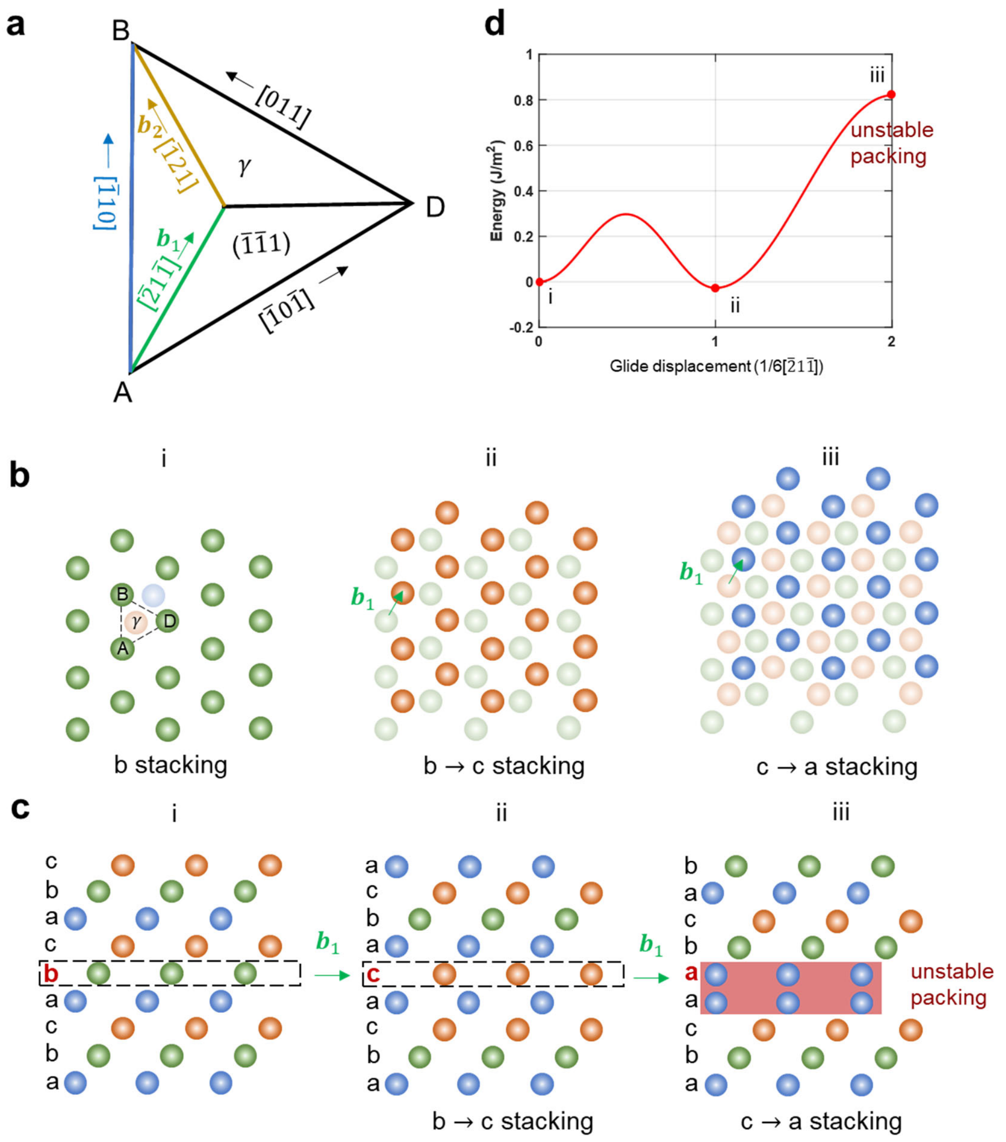

Supplementary Fig. 13 | Partial dislocation glide-induced stacking sequence change and deactivation of Frank-Read source. a, Illustration of slip systems on the ABD slip plane in the Thompson tetrahedron. **b** and **c**, Stacking sequence variation caused by passage of leading partial dislocation, $\boldsymbol{b}_1$ ([$\bar{2}1\bar{1}$]). (i), the perfect stacking sequence of abc…abc. (ii), after the glide of partial dislocation, the stacking b transforms into c stacking, forming a stacking fault. (iii), if the partial glides again, the c stacking will transform into a stacking. The a-a stacking is energetically unfavorable, making the second slippage of partial prohibited; hence the source is deactivated. Here, (**b**) viewed normal to the slip plane ($\bar{1}\bar{1}1$) (i.e., ABD), and (**c**) along the [011] (i.e., DB) direction. **d**, Energy variation with partial glide shows the unstable packing after glide of two leading partials .

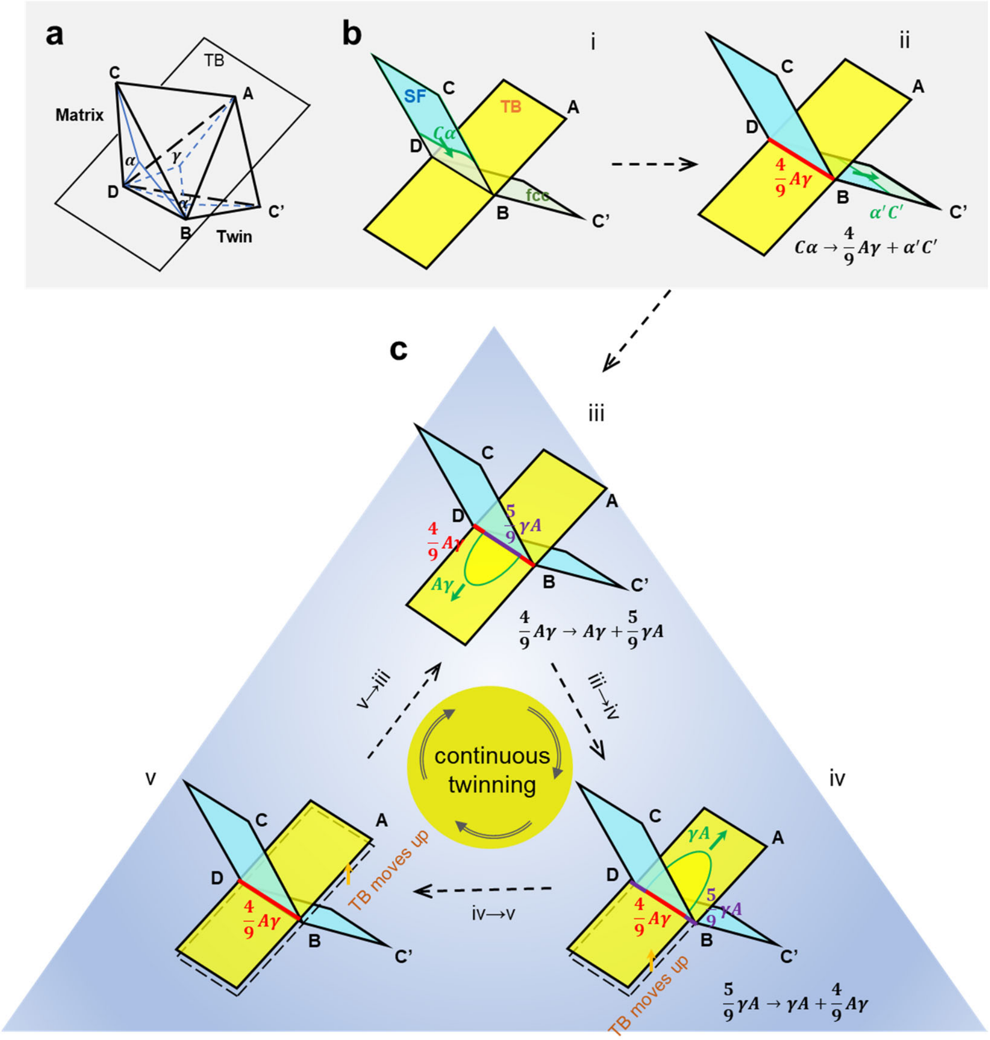

Supplementary Fig. 14 | Sustainable twinning mechanism. a, Illustration of double Thompson tetrahedra and the twin boundary (TB). **b**, An inclined partial dislocation Cα glides on BCD towards TB (i). The collision emits a partial α′C’ gliding on the mirror slip plane, B’C’D’, leaving behind a sessile partial dislocation 4/9Aγ in the intersection of SF and TB (ii). **c**, Continuous twinning dislocations nucleation. The sessile partial dislocation 4/9Aγ emits into a glissile twining partial Aγ, followed by the formation of immobile partial dislocation 5/9γA at the location. iv, the sessile 5/9γA dislocation emits twining dislocation partial γA, enabling the complete twin boundary to migrate upward by one atomic layer. v, the process creates the same configuration and a sessile partial dislocation 4/9Aγ in the intersection. By repeating iii, iv and v, continuous twinning occurs on the consecutive layers.

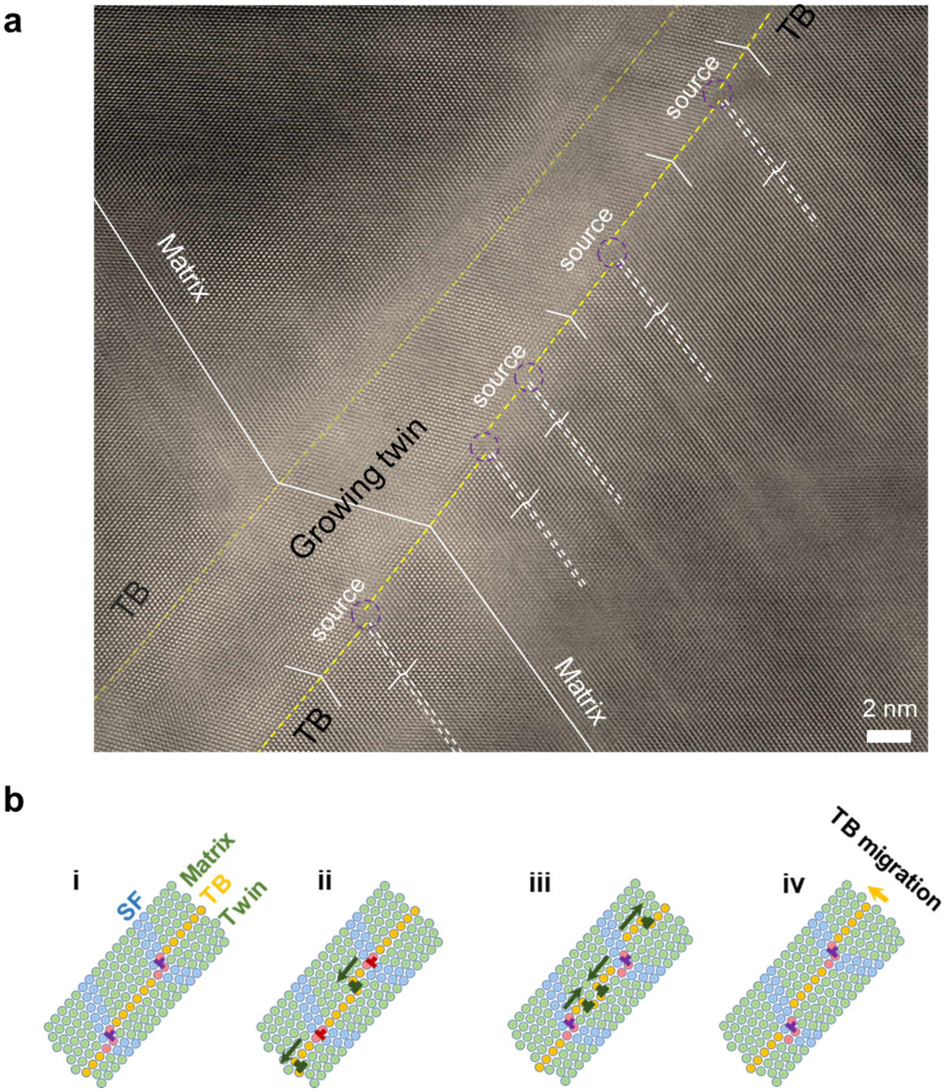

Supplementary Fig. 15 | Dislocation sources at SF-TB junctions and TB migration. a, Atomic-resolution HAADF-STEM image shows a deformation twin with concentrated SF-TB junctions and steps along the twin boundary. The dense twinning sources collectively drive TB migration and promote rapid twin growth. **b**, Atomic schematic description of rapid twin boundary migration from dynamic dislocation source: i, the schematic illustration of SF-TB junctions and steps. ii, partial dislocations decompose from twinning source and glide on left side. iii, partial dislocations glide on right side. iv, twin boundaries move up for 1 layer.

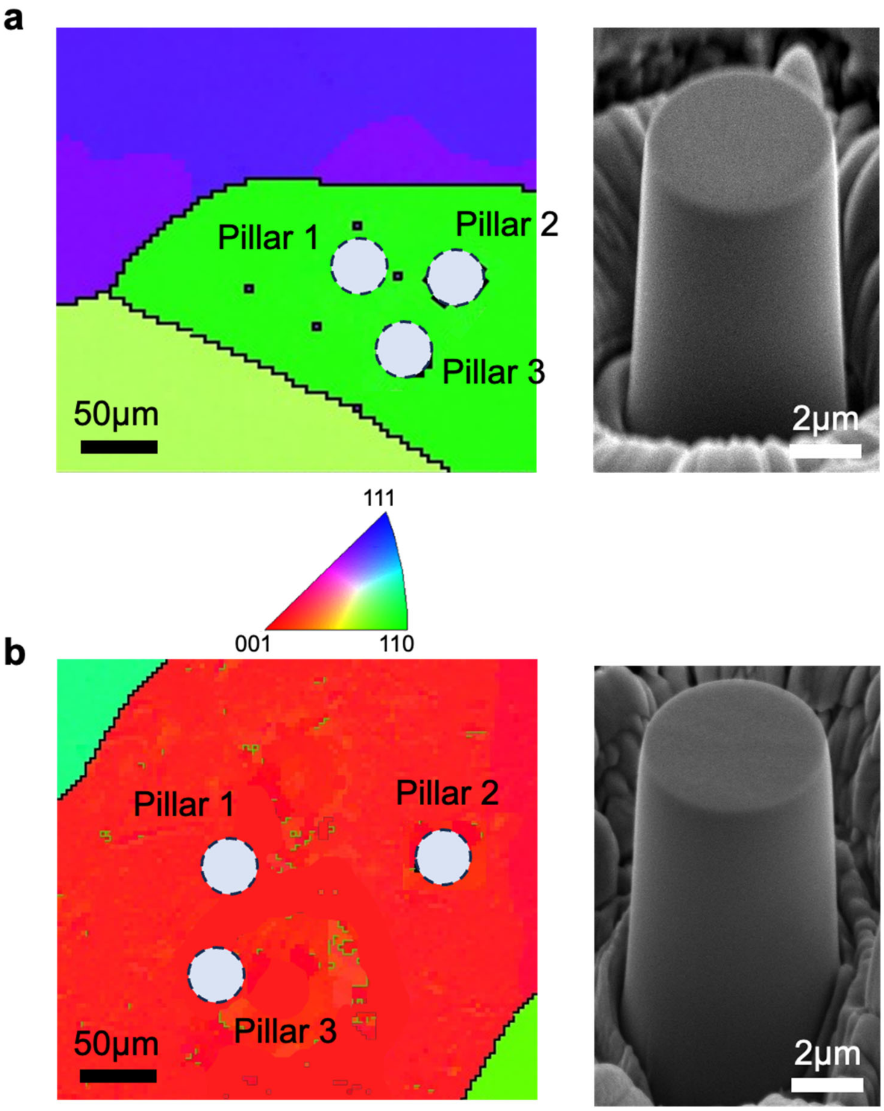

Supplementary Fig. 16 | EBSD maps showing crystallographic orientations of aged CrCoNi MEA and the single-crystal micropillar fabricated within targeted grains. a, represents the fabrication of [110]-oriented micropillars in aged MEA. **b**, shows the [100]-oriented micropillars in aged MEA.

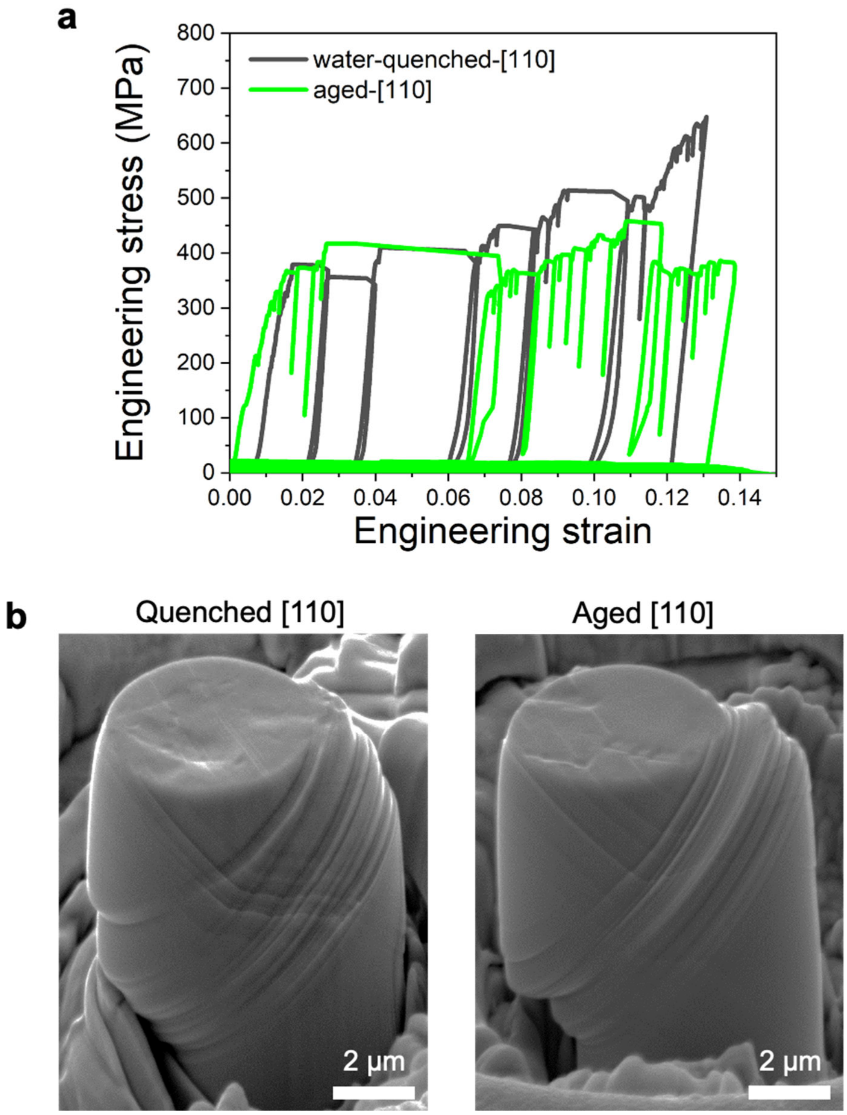

Supplementary Fig. 17 | Comparison study of serrated plastic flow and deformation morphology of quenched and aged micropillars of [110] orientation. a, Stress-strain curves of [110]-oriented micropillars of water-quenched and aged CrCoNi alloys. Both aged and quenched pillars exhibit a large stress drop event. **b**, SEM images showing the deformation surface morphology of the pillars after compression.

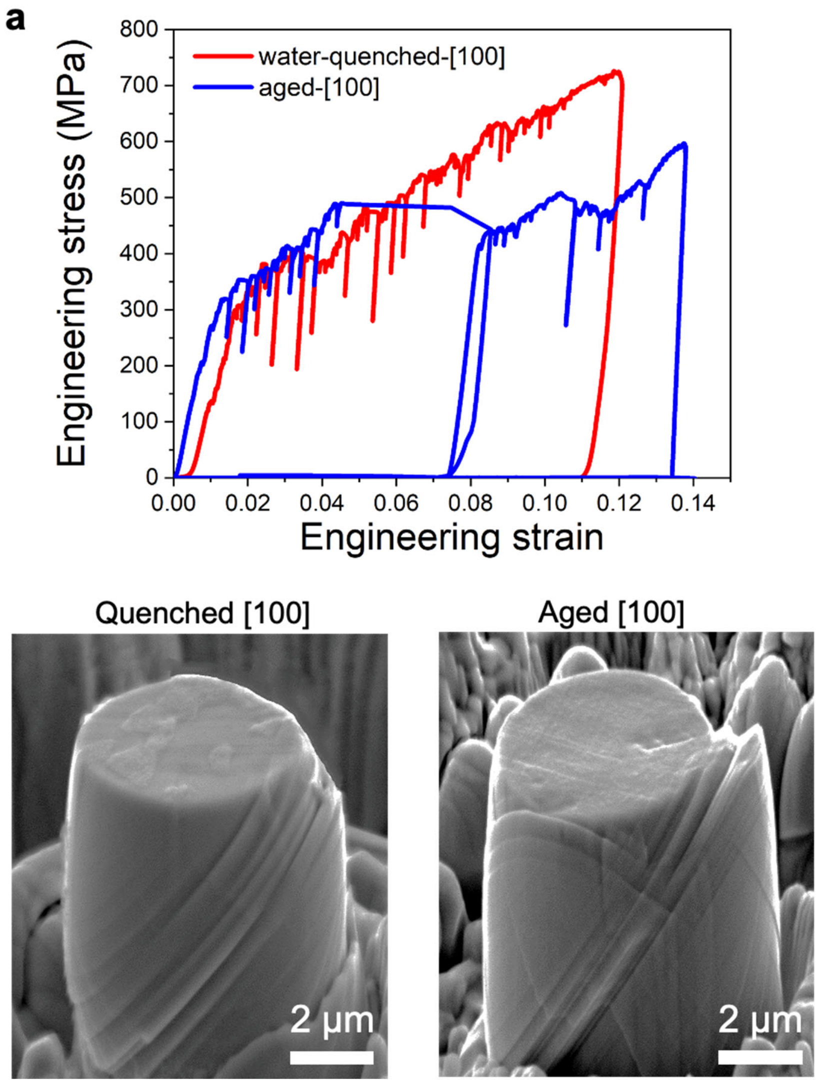

Supplementary Fig. 18 | Comparison study of serrated plastic flow and deformation morphology of quenched and aged micropillars of [100] orientation. a, Stress-strain curves of [100]-oriented micropillars of water-quenched and aged CrCoNi alloys. Compared to the quenched sample, the aged pillar exhibits large stress drop events. **b**, SEM images of deformation surface morphologies reveal the enhanced deformation localization in aged pillar.

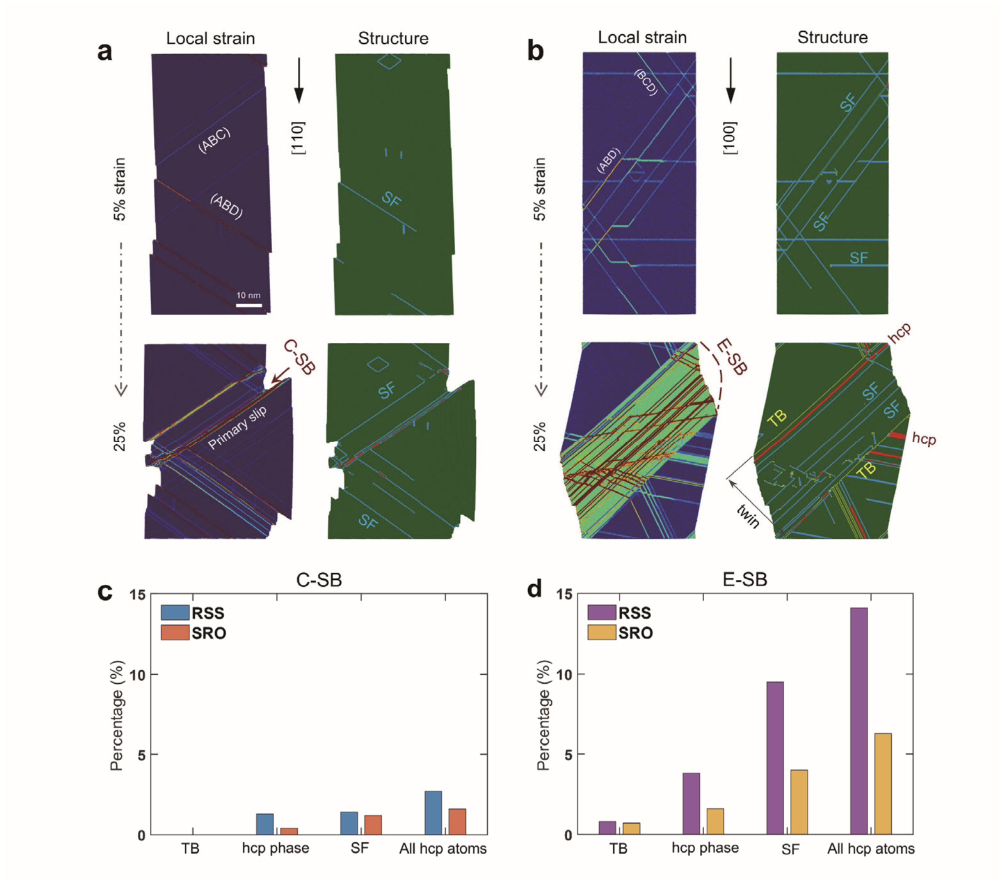

Supplementary Fig. 19 | Local plastic strain progression and microstructural evolution in aged nanopillar with short-range order. a and **b**, Evolutions of local plastic strain and microstructure with increasing strain for aged pillars with orientations of [110] and [100], respectively. The structure is depicted as follows: green color represents the fcc structure, yellow denotes TB, blue indicates SF, and red for the hcp phase. **c**, Compares the atomic fractions of TB, SF, and the hcp phase in the [110]-oriented pillar with SRO to its random solid solution (RSS) counterpart, both deformed at 25% strain. **d**, The similar comparison between SRO and RSS pillars, both deformed at 25% strain and oriented in [100] direction. In (c) and (d), the atomic strain and structure type are analyzed for the entire deformed system, which consists of 14 million atoms.

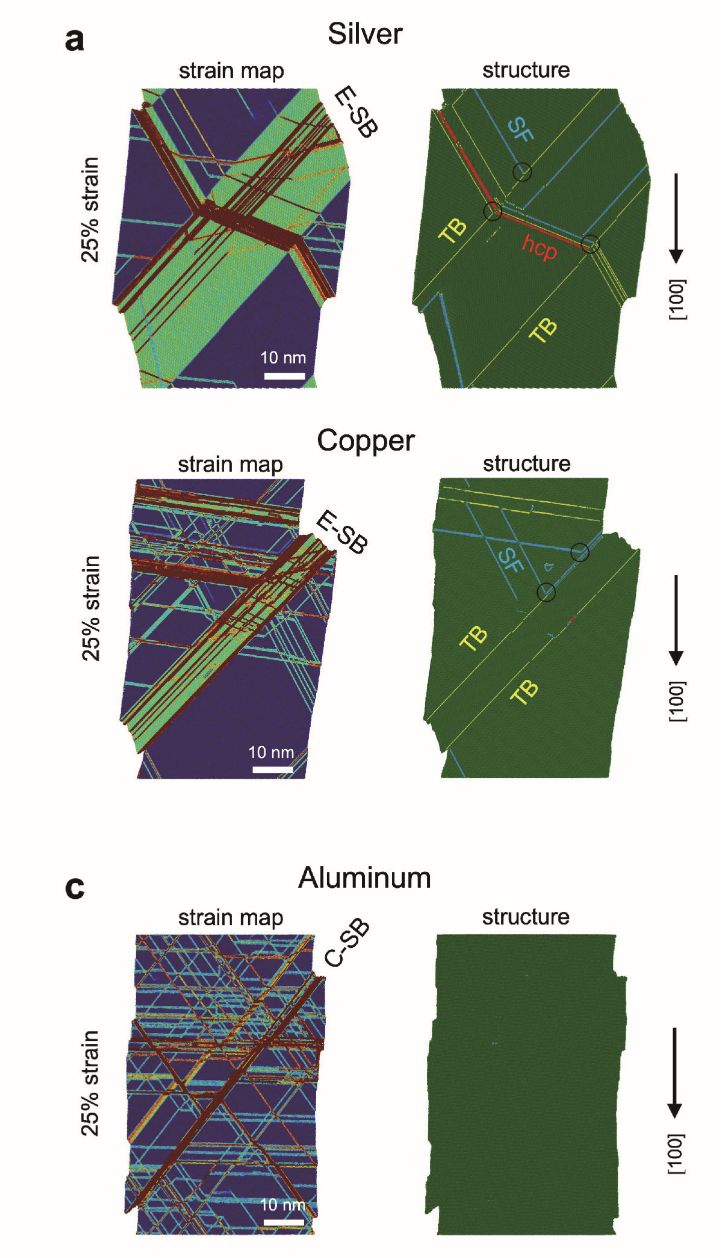

Supplementary Fig. 20 | The commonality of the E-SB mechanisms. Process of successive new source generation at the SF-TB junction, leading to E-SB formation, occurs in a group of low stacking fault energy fcc materials such as Ag, Cu, and CrCoNi, but not in Al. **a**, Local strains and deformation structures of Ag and Cu showing the E-SB and the corresponding dislocation sources at SF-TB junction. **c**, Al only shows C-EB due to perfect dislocation mechanism. The structure is depicted as follows: green color represents the fcc structure, yellow denotes TB, blue indicates SF, and red represents the hcp phase.

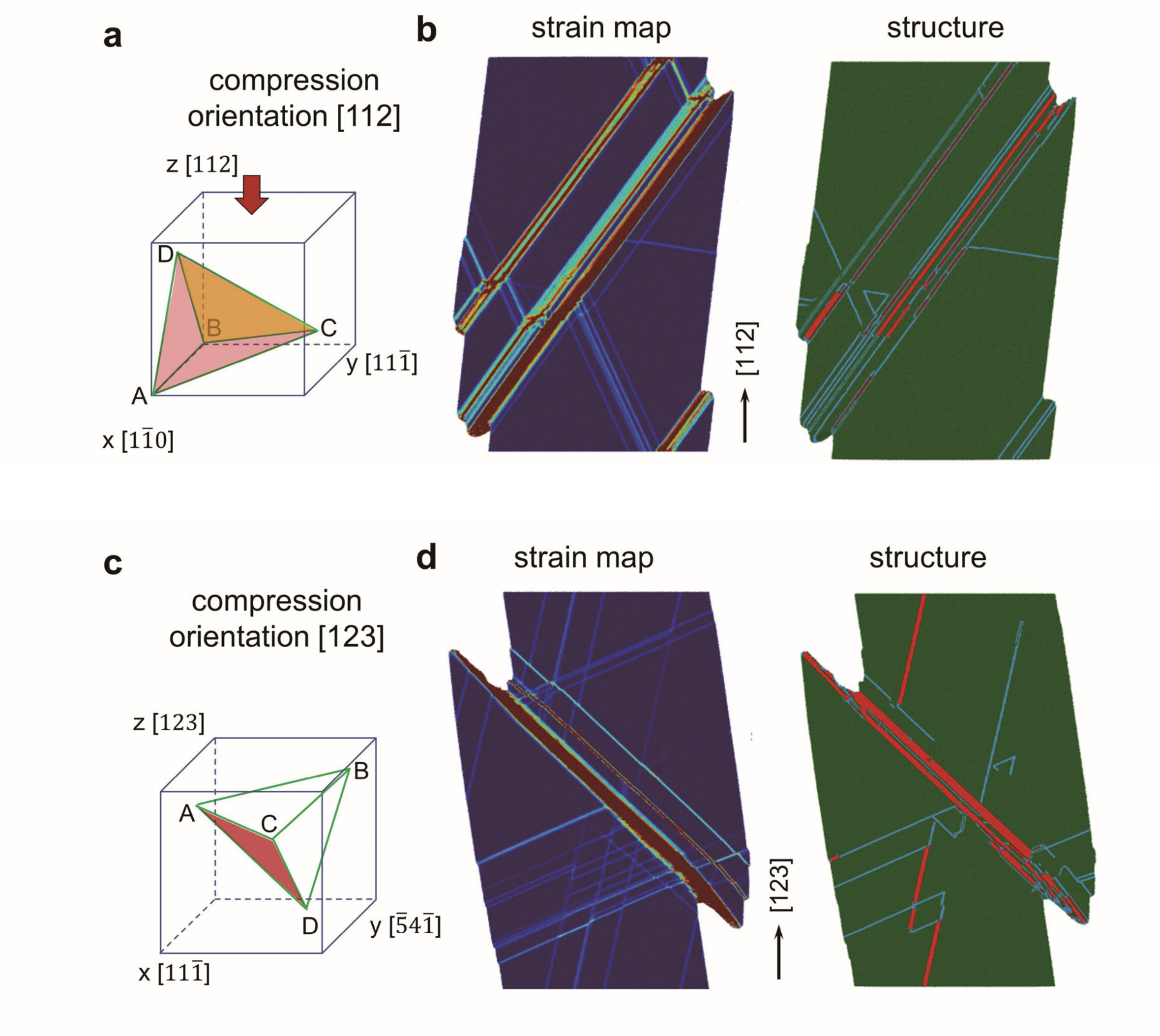

Supplementary Fig. 21 | Deformation behavior and slip banding in [112]-oriented and [123]-oriented pillars. **a**, The system has two activated slip planes (non-coplanar) with primary full dislocation mechanism. **b**, Strain map and deformation structure show the formation of C-SB in [112]-oriented pillar. **c,** The system has one activate slip system on ACD plane with primary full dislocation mechanism in [123]-oriented pillar. **d**, Strain map and deformation structure show formation of C-SB.

Supplementary Table 1 | Schmid factors of possible slip systems for [110]-oriented crystal.

	Full dislocations		Partial dislocations	
Slip plane	Slip direction	Schmid factor	Slip direction	Schmid factor
ABC (111)	AC $[01\bar{1}]$	0.4082	LP Aδ $[\bar{1}2\bar{1}]$	0.2357
			TP δC $[11\bar{2}]$	0.4714
	BC $[10\bar{1}]$	0.4082	LP Bδ $[2\bar{1}\bar{1}]$	0.2357
			TP δC $[11\bar{2}]$	0.4714
	AB $[\bar{1}10]$	0	LP Aδ $[\bar{1}2\bar{1}]$	0.2357
			TP δB $[\bar{2}11]$	-0.2357
ABD $(\bar{1}\bar{1}1)$	AD $[\bar{1}0\bar{1}]$	0.4082	LP Aγ $[\bar{2}1\bar{1}]$	0.2357
			TP γD $[\bar{1}\bar{1}\bar{2}]$	0.4714
	BD $[0\bar{1}\bar{1}]$	0.4082	LP Bγ $[1\bar{2}\bar{1}]$	0.2357
			TP γD $[\bar{1}\bar{1}\bar{2}]$	0.4714
	AB $[\bar{1}10]$	0	LP Aγ $[\bar{2}1\bar{1}]$	0.2357
			TP γB $[\bar{1}21]$	-0.2357
ACD $(1\bar{1}\bar{1})$	AD $[\bar{1}0\bar{1}]$	0	LP Aβ $[\bar{1}1\bar{2}]$	0
			TP βD $[\bar{2}\bar{1}\bar{1}]$	0
	AC $[01\bar{1}]$	0	LP Aβ $[\bar{1}1\bar{2}]$	0
			TP βC $[12\bar{1}]$	0
	CD $[\bar{1}\bar{1}0]$	0	LP Cβ $[\bar{1}\bar{2}1]$	0
			TP βD $[\bar{2}\bar{1}\bar{1}]$	0
BCD $(\bar{1}1\bar{1})$	BC $[10\bar{1}]$	0	LP Bα $[1\bar{1}\bar{2}]$	0
			TP αC $[21\bar{1}]$	0
	BD $[0\bar{1}\bar{1}]$	0	LP Bα $[1\bar{1}\bar{2}]$	0
			TP αD $[\bar{1}\bar{2}\bar{1}]$	0
	CD $[\bar{1}\bar{1}0]$	0	LP Cα $[\bar{2}\bar{1}1]$	0
			TP αD $[\bar{1}2\bar{1}]$	0

Supplementary Table 2 | Schmid factors of possible slip systems for [100]-oriented crystal.

	Full dislocations		Partial dislocations	
Slip plane	Slip direction	Schmid	Slip direction	Schmid factor
ABC (111)	BA $[1\bar{1}0]$	0.4082	LP Bδ $[2\bar{1}\bar{1}]$	0.4714
			TP δA $[1\bar{2}1]$	0.2357
	BC $[10\bar{1}]$	0.4082	LP Bδ $[2\bar{1}\bar{1}]$	0.4714
			TP δC $[11\bar{2}]$	0.2357
	AC $[01\bar{1}]$	0	LP Aδ $[\bar{1}2\bar{1}]$	-0.2357
			TP δC $[11\bar{2}]$	0.2357
ABD $(\bar{1}\bar{1}1)$	AD $[\bar{1}0\bar{1}]$	0.4082	LP Aγ $[\bar{2}1\bar{1}]$	0.4714
			TP γD $[\bar{1}\bar{1}\bar{2}]$	0.2357
	AB $[\bar{1}10]$	0.4082	LP Aγ $[\bar{2}1\bar{1}]$	0.4714
			TP γB $[\bar{1}21]$	0.2357
	DB [011]	0	LP Dγ [112]	-0.2357
			TP γB $[\bar{1}21]$	0.2357
ACD $(1\bar{1}\bar{1})$	DA [101]	0.4082	LP Dβ [211]	0.4714
			TP βA $[1\bar{1}2]$	0.2357
	DC [110]	0.4082	LP Dβ [211]	0.4714
			TP βC $[12\bar{1}]$	0.2357
	CA $[0\bar{1}1]$	0	LP Cβ $[\bar{1}\bar{2}1]$	-0.2357
			TP βA $[1\bar{1}2]$	0.2357
BCD $(\bar{1}1\bar{1})$	CB $[\bar{1}01]$	0.4082	LP Cα $[\bar{2}\bar{1}1]$	0.4714
			TP αB $[\bar{1}12]$	0.2357
	CD $[\bar{1}\bar{1}0]$	0.4082	LP Cα $[\bar{2}\bar{1}1]$	0.4714
			TP αD $[\bar{1}\bar{2}\bar{1}]$	0.2357
	BD $[0\bar{1}\bar{1}]$	0	LP Bα $[1\bar{1}\bar{2}]$	-0.2357
			TP αD $[\bar{1}\bar{2}\bar{1}]$	0.2357

Supplementary Note 1 | Deformation behavior of [110]- and [100]-oriented micropillar

We selected grains with specific orientations of [110] and [100] based on EBSD mapping (Supplementary Fig. 1). To ensure exact crystallographic orientations of [110] and [100] in all micropillars, we employed FIB to fabricate single-crystal micropillars with a diameter of 5 μm, as confirmed in Supplementary Fig. 1 [1,2]. During in-situ compression tests inside a SEM, we observed distinct stress-strain responses and deformation morphologies of [110]- and [100]-oriented micropillar (Supplementary Fig. 2 and 7, respectively). Notably, evident strain bursts and large stress drops (avalanches) are observed in the plastic flow regime of [110]-oriented micropillar (Supplementary Fig. 2a-b). These stress drops were accompanied by the appearance of distinct surface steps caused by slip (Supplementary Fig. 2c). Intriguingly, the ultra-large stress drop events are substantially diminished in the plastic deformation of [110]-oriented micropillar (Supplementary Fig. 7a-b), and the surface morphology exhibits a ribbon-like distribution with extended slip bands (E-SB) rather than sharp surface steps of confined slip band (C-SB) (Supplementary Fig. 7c).

Supplementary Note 2 | Rapid twinning mechanism in extended slip band (E-SB)

We proposed a twining mechanism from dislocation reactions and dynamical dislocation source generation. Illustrating in Thompson tetrahedron [3] (Supplementary Fig. 14a), when an inclined partial dislocation Cα gliding on the BCD plane collides with the twin boundary, a dislocation reaction occurs at the intersection of glide plane and twin boundary (Supplementary Fig. 14b i). The reaction produces a glissile partial dislocation α′C' gliding on the mirror slip plane, B'C'D', leaving behind a sessile partial dislocation 4/9Aγ in the intersection (Supplementary Fig. 14b ii). Under the applied stress, a dislocation reaction can further occur as 4/9Aγ→Aγ+5/9γA, where the sessile partial dislocation 4/9Aγ splits into a glissile twining dislocation Aγ, followed by the formation of immobile partial dislocation 5/9γA at the source. The partial dislocation Aγ glides on one side of the slip plane ABD, moving the twin boundary upward by one atomic layer (Supplementary Fig. 14c iii). The remaining partial dislocation undergoes a reaction as 5/9γA→γA+4/9Aγ, in which the emitted partial dislocation γA glides on the opposite side of the slip plane ABD, enabling the twin boundary on this side to migrate upward by one atomic layer (Supplementary Fig. 14c iv). After this, the TB moved up to its neighboring layer. Interestingly, the process creates the same configuration and a sessile partial dislocation 4/9Aγ in the intersection (Supplementary Fig. 14c v), which enables respectively twinning dislocation creation on the consecutive layers. This proposed twining process and atomic structures are consistent with our experimental observation, as shown in Fig. 5e and Supplementary Fig. 15, where twining occurs at the intersection of planar defect SF and TB.

Supplementary Note 3 | Deformation structure and slip bands in quenched and aged alloys

We fabricated single-crystal micropillars with a diameter of 5 μm from aged CrCoNi using FIB to ensure identical crystallographic orientations of [110] and [100] for all fabricated micropillars (Supplementary Fig. 16). Subsequently, in-situ compression tests were also conducted on the same oriented micropillars of aged CrCoNi as those of the quenched alloy. The comparisons of deformation behavior for both [110] and [100] orientations are presented in Supplementary Fig. 17 and 18, respectively. The stress-strain curves (Supplementary Fig. 17a) show that both [110]-oriented pillars (aged and quenched) exhibit similar ultra-high stress drops. In contrast, for [100]-oriented pillars, the aged pillar shows higher stress drops accompanied by more localized deformation bands (Supplementary Fig. 18).

Atomistic simulations of aged pillars with short-range order (SRO) were carried out to reveal microstructure evolution under compression (Supplementary Fig. 19). Similar to the pillars with random solid solution (RSS), the SRO systems develop C-SB and E-SB for compression along [110] and [100] directions, respectively. Unlike deformed RSS systems, the deformation microstructures in SRO pillars exhibit a considerable decrease in planar defects, including SF, TW, and hcp phase (Supplementary Fig. 19c-d). This reduction presumably originates from the SRO-induced increase in stacking fault energy, which suppresses the partial dislocation mechanism. It can be seen from the statistical distributions of the planar defect density in [110]-oriented and [100]-oriented pillars. Because [100]-oriented pillar has preferential partial dislocation slip, its reduction of SFs and hcp phases in the SRO system is more pronounced than that in the [100]-oriented pillar that operates full dislocation.

Supplementary Note 4 | Dislocation nucleation stress from Frank-Read source, surface step, and surface

To determine whether dislocation nucleation originates from the sample surface or within the pillar interior, we performed atomistic modeling to compare the dislocation nucleation stresses across three scenarios: free surface, surface step, and Frank-Read source.

We created three perfect pillars with diameters of 22 nm, 32 nm, and 42 nm, each with an aspect ratio of 2.5, to study dislocation nucleation stress at the free surface. These pillars contain approximately 2 million, 6 million, and 14 million atoms, respectively. To calculate the nucleation stress in the presence of a surface step, we used a perfect pillar with a 32 nm diameter and introduced surface steps. Three step sizes were generated by shifting the pillar above the (111) slip plane along the [110] direction by a few Burgers vectors. The ratios of surface step size to pillar diameter, 0.01, 0.04, and 0.09, are considered. For dislocation nucleation stress at different sized Frank-Read sources, we inserted dislocation sources with

varying pinning distances, specifically 3, 6, and 9 nm. For the three groups of system, uniaxial compressive deformation is applied to the nanopillars at a constant engineering strain rate of 5×10^{7} s^{-1} along z-axis and a temperature of 300 K using a Langevin thermostat (Methods). The yielding stresses, at which the dislocation source is activated and plastic deformation occurs, were extracted and are presented in Supplementary Fig. 6 (for [110] orientation) and Fig. 9 ([100] orientation).

For example, the results shown in Supplementary Fig. 9 reveal that the stress required to activate dislocations is lowest for pillars containing an interior defect source. Specifically, when the pinning point distance in the Frank-Read source increases from 3 to 6 and 9 nm, the nucleation stress decreases significantly from approximately 1.7 to 0.9 and 0.5 GPa, respectively. In contrast, the activation stress for a pillar with a free surface is notably higher, around 2.5 GPa. These findings suggest that dislocations are more likely to nucleate from the bulk interior at the source, where the nucleation stress is substantially lower compared to that required for surface nucleation, even in the presence of surface steps.

Supplementary References

1 Uchic, M. D., Dimiduk, D. M., Florando, J. N. & Nix, W. D. Sample Dimensions Influence Strength and Crystal Plasticity. *Sci* **305**, 986-989, 3 (2004).

2 Dimiduk, D. M., Uchic, M. D. & Parthasarathy, T. A. Size-affected single-slip behavior of pure nickel microcrystals. *Acta Mater.* **53**, 4065-4077, (2005).

3 Zhu, Y. T. *et al.* Dislocation–twin interactions in nanocrystalline fcc metals. *Acta Mater.* **59**, 812-821, (2011).
